# An mHealth Intervention to Improve Pre-Exposure Prophylaxis Knowledge Among Young Black Women in Family Planning Clinics: Development and Usability Study

**DOI:** 10.2196/37738

**Published:** 2022-07-28

**Authors:** Amy K Johnson, Sadia Haider, Katie Nikolajuk, Lisa M Kuhns, Emily Ott, Darnell Motley, Brandon Hill, Lisa Hirschhorn

**Affiliations:** 1 Division of Adolescent and Young Adult Medicine Ann & Robert H Lurie Children's Hospital of Chicago Chicago, IL United States; 2 Feinberg School of Medicine Northwestern University Chicago, IL United States; 3 Department of Obstetrics & Gynecology Rush University Chicago, IL United States; 4 Department of Medicine Chicago Center for HIV Elimination University of Chicago Chicago, IL United States; 5 Howard Brown Health Chicago, IL United States

**Keywords:** mHealth, adolescent health, young Black women, pre-exposure prophylaxis, HIV, mobile health, PrEP, mobile app

## Abstract

**Background:**

Young Black women between the ages of 18 and 24 years are disproportionately impacted by HIV, yet they have a low self-perception of HIV risk and limited exposure to prevention strategies. Pre-exposure prophylaxis (PrEP) is a safe and effective biomedical HIV prevention strategy for those at risk for HIV infection, but uptake has been slow among cisgender women. Family planning clinics are a primary source of health care access for young women, providing an ideal opportunity to integrate PrEP information and care into existing clinic practices.

**Objective:**

The aim of this study was to use a multistage, community-engaged process to develop a mobile health app and to evaluate the feasibility and acceptability of the app.

**Methods:**

Using user-centered design, the *In the Loop* app was developed in collaboration with a community advisory board of young Black women. This study employed a multistage design, which included community-engaged app development, user testing, and evaluation of the app’s feasibility and acceptability. A pre- and postdesign was used to assess the impact of the app on PrEP knowledge immediately after app use. Descriptive statistics (eg, mean, SD, and percentage values) were used to describe the sample, and Wilcoxon matched-pairs signed-ranks test was used to detect changes in PrEP knowledge before and immediately after using the app.

**Results:**

A total of 50 sexually active, young Black women, aged 18-24 (mean 21, SD 1.9) years, were enrolled in this study. Analysis comparing scores before and immediately after use of the app revealed a significant increase in PrEP content knowledge scores on a 7-item true or false scale (*z*=–6.04, *P*<.001). Overall, participants considered the *In the Loop* app feasible and acceptable to use while waiting for a family planning visit. The majority of participants (n=46, 92%) agreed that they would recommend *In the Loop* to friends to learn more about PrEP. Participants rated the overall quality of the app 4.3 on a 1-5 scale (1=very poor and 5=very good). Of 50 participants, 40 (80%) agreed that the app was easy to use, and 48 (96%) agreed that they found the information in the app easy to understand. Finally, 40 (80%) agreed that they had enjoyed using the app while waiting for their family planning visit.

**Conclusions:**

Our findings suggest that young Black women waiting for family planning visits found the *In the Loop* app to be feasible and acceptable. This study demonstrates the value of engaging young Black women in the app design process. As family planning clinics are a primary source of health care access for young women, they provide an ideal setting to integrate PrEP information and care into existing clinic practices. Next steps in the development of the *In the Loop* app include implementing user-suggested improvements and conducting efficacy testing in a randomized controlled trial to determine the app’s impact on PrEP uptake.

## Introduction

Approximately 23% of all people living with HIV in the United States are cisgender women, and women accounted for 19% of new infections in 2018 [1]. The majority (87%) of HIV infections among women are due to heterosexual sex [1]. Black women are disproportionately impacted by HIV, with an 18-fold higher risk of acquiring HIV compared to White women [2]. In Chicago, 85.9% of new HIV infections among women occur among non-Hispanic Black women [3]. In particular, young Black women aged 18-24 years often have multiple co-occurring risk factors (eg, high HIV density in sexual networks, concurrent sexual partners, experiences of violence, substance use, and sex partners with incarceration history), yet they have a low self-perception of HIV risk, complicating HIV prevention efforts [3-7].

Pre-exposure prophylaxis (PrEP) is a safe and effective biomedical HIV prevention strategy for those at risk for HIV infection, including young Black women [8,9]. PrEP has the advantage over other prevention interventions of allowing women to have autonomy and control over their sexual health because it does not require a sexual partner’s permission or cooperation for use [10]. However, women make up a disproportionately low percentage of PrEP users in the United States [11]. The Centers for Disease Control and Prevention estimates that 468,000 women in the United States are eligible for PrEP, but only 19,000 women have ever been prescribed PrEP [11-13]. From 2014 to 2016, only 2% of women who have indication for PrEP received a prescription, and women accounted for less than 5% of all PrEP users in 2016 [10]. Chicago is a leading city in PrEP implementation, but uptake of PrEP among women is extremely low. For example, of the patients who have initiated PrEP in sexually transmitted infections (STI) clinics of the Chicago Department of Public Health, only 1.5% are women [2,14]. Known individual-level barriers to PrEP uptake among women include low rates of PrEP knowledge, low perceived risk of HIV acquisition, and lack of awareness of how and where to access PrEP [2,14]. Increasing knowledge, risk perception, awareness, and uptake of PrEP among young Black women who may be at an increased risk for HIV are key public health interventions that have the potential to reduce new HIV infections [15].

Family planning clinics are primary sources of health care access for young women, providing an ideal opportunity to integrate PrEP information and care into existing clinic practices [16,17]. A total of 60% of women, including young Black women, consider family planning clinics their primary source of medical care, with 40% indicating it is their only source of health care [17]. In 2018, Title X family planning clinics served over 3.9 million patients, most of whom were young (63%), female (87%), and had low income (65%) [18]. Over 1 million HIV tests were performed in Title X clinics in 2018, establishing a precedent for receiving HIV prevention services at family planning clinics [18]. Optimizing PrEP awareness via integration with family planning services has the potential to increase PrEP uptake and impact incidence on a population level, but it has not yet been fully explored or implemented.

The ubiquitous nature of new digital media, characterized by its adaptability and interactivity, offers opportunities to disseminate confidential information to adolescents in a relevant, youth-friendly format. Mobile health (mHealth) approaches have been proven to be most impactful when integrated into existing health system functions, rather than as stand-alone solutions [19,20]. Given the limited provider time per patient, brief theory-driven mHealth intervention models delivered in family planning clinics waiting rooms have the potential to aid patients in increasing knowledge, informing decision-making, and framing questions to providers [21-23].

Researchers have identified the lack of tailored apps for minority communities as an ongoing challenge in digital media intervention [24,25]. To date, no studies have been conducted to design, target, and promote an mHealth intervention to enhance HIV risk assessment, increase PrEP knowledge, and influence PrEP uptake among young Black women within a family planning setting. Patients prefer health information to be interactive [26]; a waiting room–based mHealth intervention uses a current missed opportunity to enhance HIV risk assessment and provide patient education about PrEP, and it has the potential for high impact [22,23]. This pilot study is the first step in designing and implementing an mHealth HIV prevention and PrEP promotion intervention tailored for young Black women.

## Methods

This pilot study employed a multistage design that included community-engaged app development, user testing, and evaluation of the app’s feasibility and acceptability.

### Ethics Approval

All procedures were approved by the Institutional Review Board at Lurie Children’s Hospital and the University of Chicago (2018-2189).

### Setting

The 2017 rate (27.9 per 100,000 population) of HIV infection diagnoses in Chicago is approximately 2.5 times higher than the national rate, and the prevalence rate for Chicago (827.9 per 100,000 population) is nearly 3 times the national rate [27]. Community areas on the West and South sides of Chicago represent a concentrated HIV epidemic, with HIV prevalence consistently over 5% [3,27]. The study recruitment site was a Planned Parenthood Illinois clinic that is located centrally within the city and serves predominantly patients of color.

### In the Loop App Prototype Development

A community advisory board (CAB) of 9 young Black women was formed to guide and tailor content of the app prototype. CAB participants were recruited through partner agencies, email listservs, and flyers posted throughout the community. Recruitment flyers included information about the project, participation expectations, and learning opportunities (ie, the app design process). Our goal was to have a group of 8-10 young women who met the inclusion criteria for the larger pilot study to participate in the CAB. CAB members were provided with a US $50 incentive for each 2-hour meeting they attended.

Guided by user-centered design and the information systems research frameworks, the study team conducted relevance, rigor, and design cycles to refine and adapt an existing prototype app called *miPrEP* [28]. The *miPrEP* app was developed to increase PrEP knowledge and engagement among young Black men who have sex with men [29]. The CAB conducted 4 design sprints over a 3-month period; sessions were guided by generating app content and approving mock-ups (ie, iterative design process). In the rigor cycle, the CAB members met and reviewed the available components of the *miPrEP* app. The *miPrEP* app contained basic PrEP information, as well as a video about how PrEP works in the body. The study team identified and presented additional health-based apps to the CAB for review. Existing content and desired features of an adapted sexual health app were discussed and ranked. Finally, features and content were identified for inclusion and tailoring for the *In the Loop* prototype. In the design cycle, a low fidelity prototype was developed. Paper and pencil mock-ups were used to design the visual assets, video content, and text materials. The prototype of *In the Loop* was created using paper models to represent each intervention component and a Google slide deck to demonstrate app navigation and flow. All elements were iteratively revised with the CAB until there was consensus on the content, quantities, and the form of the app.

### User Testing and Pilot Evaluation

Potential participants were recruited from a Planned Parenthood clinic waiting room and, if interested and eligible, they completed informed consent and the study procedures. Participants were eligible if they (1) reported being cisgender women; (2) identified as African American or Black; (3) were English speakers; (4) were aged 18-24 years (inclusive); (5) reported vaginal or anal sex with a male partner within the past 6 months; (6) were seeking testing or treatment for a sexually transmitted infection, or seeking contraception or abortion services; (7) self-reported being HIV-negative; (8) were not currently pregnant; and (9) were neither currently taking PrEP as HIV prevention nor intending to initiate PrEP at the current clinic appointment.

A pre- and postdesign assessment was used in this pilot study to evaluate the impact of the app on PrEP knowledge immediately after using the app. PrEP knowledge was measured via 7 true or false items that were selected based on use in previous studies with cisgender women [14,30]. Other study aims were to assess usability, including basic feasibility and acceptability of the app prototype among young Black women. Usability was assessed through open-ended questions that asked participants their opinion about the app (eg, “what did you like about using *In the Loop* while waiting for your appointment?” and “what information would you add to *In the Loop* app?”) and ways to improve the app (eg, text or graphic display, navigation, and content). Acceptability of *In the Loop* was evaluated using a series of 9 items derived from an abbreviated acceptability rating scale used in prior research [31]. Participants used a 5-point Likert scale (1=strongly disagree and 5=strongly agree) to rate the extent to which they agreed with each acceptability statement (eg, “I found the information in the app easy to understand and comprehend”). The measure was administered at the immediate postintervention assessment. Overall interest in using mHealth for sexual health was measured via a 7-item scale based on previous research with adolescents (eg, “how likely are you to download a sexual health mobile phone app?”) [32-34]. Finally, participants were asked about preferences toward emerging modalities of PrEP, specifically their interest in a vaginal ring, implant, or injection, or PrEP combined with birth control [35].

### Analysis

Descriptive statistics (eg, mean, SD, and percentage values) were used to describe the sample, and Wilcoxon matched-pairs signed-ranks test was used to detect changes in PrEP knowledge before and immediately after using the app [36]. Open-ended responses were recorded verbatim and were thematically coded by the first author (AKJ), following a standardized procedure [37]. Each open-ended response was read twice, themes were then created based on response content, and each response was coded into one of 4 main themes. If the response contained more than one theme, it was coded as having both themes.

## Results

### Participant Characteristics

**Figure 1:**
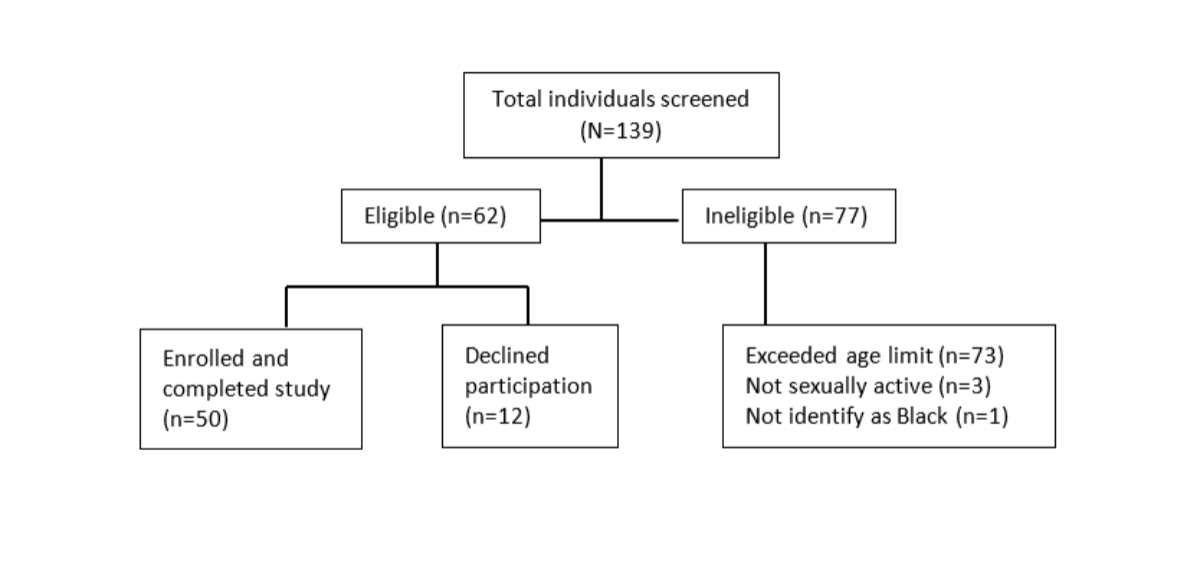
CONSORT (Consolidated Standards of Reporting Trials) flow diagram.

The *In the Loop* prototype was evaluated from July 2019 through August 2019. A total of 50 sexually active, young Black women aged 18-24 (mean 21, SD 1.9) years were enrolled in the study. Initially, a total of 139 individuals were screened, with 62 being eligible; of those eligible, 50 (81%) enrolled and completed the study visit (Figure 1). 

Of the 50 participants, 23 (46%) reported being current students, with 21 (42%) participants reporting their highest level of education as some college education, 19 (38%) as high school graduate, and 9 (18%) of them reported having completed college (Table 1). The majority of participants (n=37, 74%) were employed either full- or part-time. To characterize potential social determinants of health that impact HIV transmission, participants reported whether they had ever received government assistance (n=19, 38%) and whether they had ever been homeless (n=11, 22%).

A total of 44 (88%) participants reported identifying as straight or heterosexual, and 6 (12%) reported their sexual orientation as bisexual. In terms of sexual behavior, the majority (n=42, 84%) reported having 1 sex partner in the past 3 months. Inconsistent condom use was common, with 43 (86%) participants reporting sometimes or never using condoms during vaginal sex; 9 (18%) participants reported having sex with a person whose HIV status they did not know; 6 (12%) of them had a recent STI diagnosis, and 24 (48%) had experienced an unplanned pregnancy.

**Table 1:** Participant characteristics (N=50).

Characteristics			Values
Age, mean (SD)			21 (1.9)
**Sexual orientation, n (%)**			
	Straight	44 (88)	
	Bisexual	6 (12)	
**Hispanic or Latina ethnicity, n (%)**			
	No	47 (94)	
	Yes	3 (6)	
**Education level, n (%)**			
	Some high school	1 (2)	
	High school or General Education Diploma	19 (38)	
	Some college or Associate of Arts degree	21 (42)	
	Completed college or Bachelor of Arts degree	9 (18)	
**Current student, n (%)**			
	Yes	23 (46)	
	No	27 (54)	
**Received government aid, n (%)**			
	Yes	19 (38)	
	No	29 (58)	
	Unsure	2 (4)	
**Employment, n (%)**			
	No	13 (26)	
	Part-time	23 (46)	
	Full-time	14 (28)	
**Ever been homeless, n (%)**			
	Yes	11 (22)	
	No	39 (78)	
**Sexual behavior in the past 3 months, n (%)**			
	1 sex partner	42 (84)	
	2 or more sex partners with no overlap	6 (12)	
	2 or more sex partners with overlap	2 (4)	
**Condom use in vaginal sex, n (%)**			
	Always	6 (12)	
	Sometimes	25 (50)	
	Never	18 (36)	
**Condom use in anal sex, n (%)**			
	Always	1 (2)	
	Sometimes	4 (8)	
	Never	5 (10)	
	Did not have anal sex	40 (80)	
**Had sex with a person whose HIV status you did not know, n (%)**			
	Yes	9 (18)	
	No	41 (82)	
**Had a sexually transmitted infection, n (%)**			
	Yes	6 (12)	
	No	44 (88)	
**Unplanned pregnancies, n (%)**			
	Yes	24 (48)	
	No	26 (52)	
**How likely are you to become infected with HIV based on current behavior, n (%)**			
	Very unlikely	22 (44)	
	Unlikely	22 (44)	
	Somewhat likely	6 (12)	

### PrEP Knowledge

Prior to using the app, 30 (60%) participants reported having “never heard of PrEP” and 44 (88%) considered themselves as “very unlikely or unlikely” at risk for HIV based on their current sexual behaviors. Analysis comparing scores before and immediately after using the app revealed a significant increase in PrEP content knowledge scores on a 7-item true or false scale. Total mean scores improved from 2.8 (SD 1.2) prior to using the app to 4.9 (SD 1.4) immediately after using the app (*z*=–6.04, *P*<.001). In item-by-item analysis, 4 of 7 items showed statistically significant improvements from before to immediately after app use (Table 2). After using the app, 13 (26%) participants reported they were “very likely” to start PrEP in the next 3 months, and 46 (92%) participants agreed with the statement “PrEP is a good option for HIV prevention for young women like me.”

**Figure 2:**
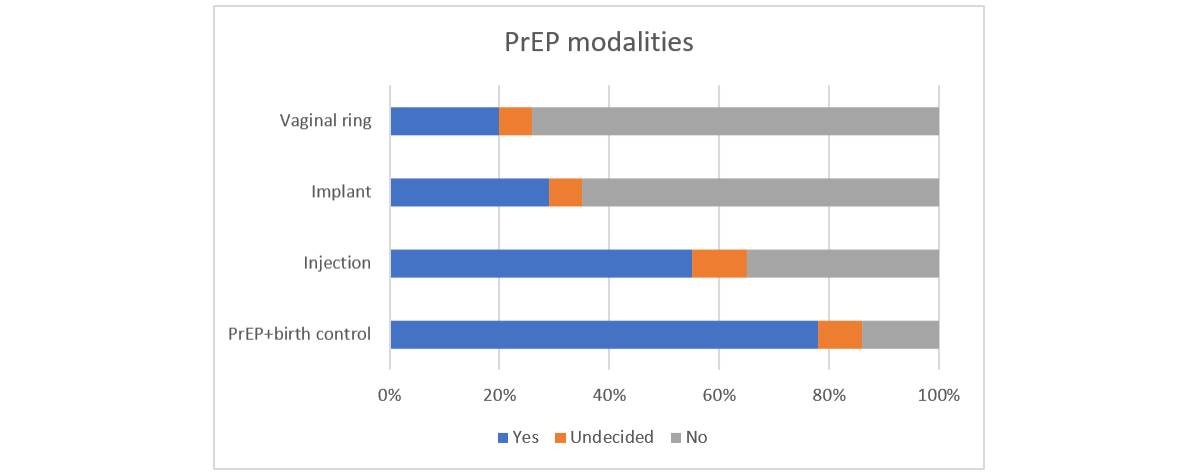
Interest in alternate PrEP modalities (N=50). PrEP: pre-exposure prophylaxis.

In the immediate postsurvey, participants were asked whether they were interested in other PrEP modalities (Figure 2), including vaginal ring, implant, or injection, or PrEP combined with birth control. The most frequently endorsed mode was a dual method option with PrEP and birth control combined; 39 (78%) participants endorsed this option.

**Table 2:** Pre-exposure prophylaxis (PrEP) knowledge before and immediately after use of the app (N=50).

True or false items	Negative ranks, n (%)	Ties, n (%)	*Z* scores
PrEP can be bought over the counter without a prescription	6 (12)	39 (78)	–0.63
PrEP needs to be taken daily in order to be effective	13 (26)	36 (72)	–3.60^a^
PrEP is only recommended for gay and bisexual men	42 (84)	7 (14)	–6.48^a^
PrEP can be used to prevent infection after being exposed to HIV	29 (58)	20 (40)	–5.39^a^
PrEP can be taken by both men and women to prevent HIV infection	4 (8)	44 (88)	–1.34
You need to be at least 18 years of age to get PrEP	11 (22)	37 (74)	–2.89^a^
Only HIV doctors can prescribe PrEP	9 (18)	37 (74)	–1.73

^a^*P*<.001.

### Technology Use for Sexual Health

When participants were asked about interest levels in using technology as part of sexual health care, 22 (44%) indicated they were very interested, and 21 (42%) reported being somewhat interested. A total of 31 (63%) indicated they were very interested in using a mobile app for sexual health education, 10 (21%) indicated they were somewhat interested, and 8 (16%) indicated no interest. Just over half (n=26, 52%) of the sample reported being very interested in receiving sexual health reminders via email (n=29, 57%) or text message (n=25, 51%). Finally, only 17 (35%) participants reported being very interested in learning about sexual health topics via internet games.

### In the Loop App Acceptability and Usability

Overall, participants considered the *In the Loop* app feasible and acceptable to use while waiting for a family planning visit. More specifically, 46 (92%) participants agreed that they would recommend *In the Loop* to friends to learn more about PrEP. Participants also reported that they liked using the app—rated 4.6 on a scale from 1 (strongly disagree) to 5 (strongly agree). Participants rated the overall quality of the app 4.3 on a scale from 1 (very poor) to 5 (very good); 40 (80%) participants agreed that the app was easy to use, and 48 (96%) agreed that they found the information in the app easy to understand. Finally, 40 (80%) agreed that they had enjoyed using the app while waiting for their family planning visit.

Participants were also asked to provide open-ended feedback on improvements that could be made to the app, which were summarized into four different themes to inform future refinement: increasing interactive components, refining app navigation, refining app aesthetics, and increasing sexual health and HIV prevention information (Table 3). Finally, participants responded to an open-ended question providing insight to their favorite parts of the app, including liking the fact that the main character is a young Black woman (“I liked that it was relatable. I liked that there was a character that looked like me” [participant ID, ie, PID 66]), that it did not require a lot of time to use (“It was quick. Wasn’t long. Wasn’t boring” [PID 15]), and that it was private or confidential (“Private on the iPad” [PID 50]).

Two functional bugs were identified in testing the app with users. The first bug caused a video to stall if the navigate back button was pushed; the second bug caused an incorrect follow-up prompt to be displayed. Both bugs, once identified, were remedied.

**Table 3:** Suggested app improvements.

Theme	Illustrative quote
Increase interactive components	“It was kind of wordy. They should highlight words, so it won’t look like a whole paragraph. The paragraph is intimidating. Should be more interactive.” [PID 71]
Refine app navigation	“Pointing out things, like for each section, so they know what they can press and where to move on, or how to leave the video.” [PID 60]
Refine app esthetics	“The blue is okay, but add more bright colors…like when you are navigating change the colors up.” [PID 82]
Increase sexual health and HIV prevention information	“Add place that people can go to get STI^a^ testing or on PrEP^b^. Like if you want to go somewhere else you put in your zipcode and go.” [PID 28] “More about mechanisms or each birth control option; how some of these birth controls are inserted; how does it work?” [PID 16]

^a^STI: sexually transmitted infection.

^b^PrEP: pre-exposure prophylaxis.

## Discussion

### Principal Findings

This study demonstrates that using an app in a family planning clinic waiting room is feasible and acceptable to young Black women. As family planning clinics are primary sources of health care for young Black women, integrating PrEP services into their practices is a critical component of comprehensive reproductive health care. Further, providing high-quality, confidential information via an mHealth app while waiting for clinic visits makes the most of a current missed opportunity. The *In the Loop* app is an innovative strategy to engage young Black women in HIV prevention.

*In the Loop* followed key tenets of developing effective mHealth interventions, including incorporating ease of modification and tailoring to the target population. Our team, in collaboration with young Black women, modified the content of an existing PrEP knowledge app and tailored it to be specific to the needs of the target population. Through user testing, we identified and addressed 2 bugs in the app. Finally, we gathered feedback on further refinements such as increasing the interactive components and refining the app navigation. The majority of participants were interested in using technology as part of sexual health care, with an app being the most popular mode of technology selected.

### Comparison With Prior Work

Cordova et al [38] designed and evaluated an mHealth intervention—*Storytelling 4 Empowerment—*to provide HIV, STI, and drug abuse prevention information to adolescents in primary care waiting rooms. In the acceptability pilot, participants reported approval of using down time while waiting for clinic visits; however, they also highlighted the need for privacy in shared spaces. Participants recommended privacy covers over tablets. In our pilot study, participants noted that the app felt private and did not raise concerns over privacy of participating in an mHealth app in a family planning clinic waiting room; nevertheless, it should be considered best practice to use privacy screens on tablets as recommended by Cordova et al [38].

In a systematic review, Chávez et al [39] highlighted the fact that apps tailored to adolescents’ specific race, ethnicity, and gender identity demonstrated stronger effects on health behavior compared to apps that were not tailored. Results from our community-engaged app development demonstrated high levels of app acceptability among the target population. Not only is the tailoring of mHealth important to success, but also the inclusion of the target population in mHealth development is critical for ensuring salience and acceptability.

In a 2020 systematic review of mHealth strategies to promote PrEP uptake, only 1 app was identified that was tailored specifically for young women; it was designed for those aged 11-14 years in Western Kenya [40,41]. No apps were identified that were tailored for young Black women in the United States. The results of the review highlight the need for mHealth strategies designed specifically to meet the needs of young Black women. We believe that our app will help to fill this gap.

### Limitations

Results of this pilot study must be interpreted in light of several limitations. First, the app was tailored exclusively for young Black women in a single urban setting, and thus our results cannot be generalized to other populations of young Black women. Further, the intervention was tailored for young Black women who have sex with men; further research is needed to tailor the intervention for young Black women who have sex with women or transgender and gender-diverse individuals. Second, the high positive rating of the app may be biased due to social desirability. We attempted to mitigate the effect of this bias by using computer-assisted questionnaires. Finally, although *In the Loop* was found to be acceptable and feasible, and to have an immediate impact on PrEP knowledge, future studies are needed to determine the sustained effect of the app on PrEP knowledge and to determine whether the app can improve PrEP uptake. Our results are limited, as they reflect prescores and immediate postscores without randomization; future studies should use randomization and assess durable effects of the intervention. Despite these limitations, this formative research may help guide future design and implementation of mHealth interventions in family planning settings, optimizing their chances for success.

### Conclusions

Overall, our findings suggest that young Black women waiting for family planning visits found *In the Loop* to be feasible and acceptable. Further, this study demonstrates the value of engaging young Black women in the design process. As family planning clinics are primary sources of health care access for young women, they provide an ideal setting to integrate PrEP information and care into existing clinic practices. mHealth approaches can be scaled rapidly with fidelity. Future studies should evaluate the efficacy and durability of the *In the Loop* app in improving PrEP knowledge and uptake among PrEP-eligible young Black women.

## Abbreviations

CAB: community advisory board
PID: participant ID
PrEP: pre-exposure prophylaxis
STI: sexually transmitted infection
